# Experiment Study on the Influence of Density and Confining Pressure on Triaxial Shear Properties of Calcareous Sand

**DOI:** 10.3390/ma16041683

**Published:** 2023-02-17

**Authors:** Hui Zhang, Huiqi Ren, Chaomin Mu, Xiangyun Wu, Kui Huang, Fei Wang

**Affiliations:** 1 State Key Laboratory of Mining Response and Disaster Prevention and Control in Deep Coal Mine, Anhui University of Science and Technology, Huainan 232001, China; 2 Defense Engineering Institute, Academy of Military Sciences, People’s Liberation Army, Luoyang 471023, China

**Keywords:** calcareous sand, relative density, confining pressure, triaxial consolidation drainage shear

## Abstract

Calcareous sand is one of the main building materials in the construction of islands and reefs, and its shear property is very important for predicting their strength and deformation. However, the correlation research on the shear properties of calcareous sand is limited. In this paper, a series of the triaxial consolidation drainage shear tests of calcareous sand with relative densities (Dr) of 70% and 90% under confining pressures of 100, 200, 400 and 800 kPa were carried out by a triaxial testing apparatus, and the effects of relative density and confining pressure on the deformation and strength characteristics of calcareous sand were analyzed. The results show that the stress–strain curves of calcareous sand show a strain softening characteristic, and both peak deviatoric stress and failure strain increase with confining pressure, but the increase in failure strain is restrained when the confining pressure is larger than 400 kPa. The initial shear modulus of calcareous sand is positively correlated with confining pressure. Additionally, the molar circular envelope of calcareous sand is linear in the range of 100~400 kPa, but it deviates from linear when confining pressure exceeds 400 kPa. The critical state line (CSL) of calcareous sand is nonlinear, with almost the same exponent for calcareous sand with different relative densities. The research results have important reference value for the foundation construction of islands and reefs.

## 1. Introduction

Calcareous sand is a special type of sand mainly composed of coral detritus and contains some other substances, such as coral algae, shells and porifera detritus [1]. It is mainly found in coral islands and reefs and their surrounding environments in tropical and subtropical marine areas, with a main component of CaCO_3_. After long-term physical, biological and chemical actions, calcareous sand forms a unique spatial structure, with distinctive characteristics such as having an irregular particle shape and multi-porosity, and being easy to break [2,3].

Most islands and reefs are far away from the mainland, and in the construction and engineering of islands and reefs, the cost of transporting materials from land is high, so calcareous sand is often used locally as a foundation material for infrastructure such as road embankments and airport runways [4,5]. Extensive research has shown that the physical and mechanical properties of marine biogenic calcareous sand are quite different from those of common terrestrial sand, and the results of existing research cannot be directly applied to calcareous sand [6,7,8,9,10,11,12,13,14,15]. Therefore, it is significant to understand the shear characteristics of calcareous sand foundation.

Some studies have been carried out on the shear properties of calcareous sand. In the direct shear test, Fahey [16] found that the yield point of calcareous sand was obvious under low confining pressure, but not obvious under high confining pressure. Ma et al. [17] studied the effects of moisture content, dry density and mineral composition on the shear properties of calcareous sand. It was found that the internal friction angle of calcareous sand increases with the increase in dry density, the apparent cohesion increases with the increase in particle size and the effect of water reduces the strength of calcareous sand. Li et al. [18] investigated the effects of axial compression, shear rate and gradation on the mechanical properties of calcareous sand. The study found that the shear strength increases with the decrease in shear rate and the increase in axial pressure and the coarse particle content. In the cyclic simple shear test, Ji et al. [19] found that the shear stress of calcareous sand is obviously lower than that of ordinary quartz sand at the stage of shear stress difference, up to 14.7%. In the triaxial test, some scholars [20,21,22,23] found that calcareous sand shows dilatancy under low confining pressure. The weakening trend of dilatancy gradually turns into shrinkage with the increase in confining pressure, and it shows absolute shear shrinkage when the confining pressure reaches more than 800 kPa [24]. Mo et al. [23] and Sun et al. [25] found that the stress–strain curve of calcareous sand gradually changed from strain softening to hardening with the increase in confining pressure. Li [24] pointed out that the specimens with low confining pressure and high relative density showed strain softening and strain hardening in samples with high effective confining pressure and low relative density. Wang et al. [26] found that the peak friction angle and critical friction angle decreased with the increase in confining pressure. Weng et al. [27] found that when the relative density of calcareous sand is greater than 70%, the internal friction angle decreased with the increase in relative density.

As reported in the above literature, the shear properties of calcareous sand are complex and affected by many factors such as density, confining pressure, shear rate, particle gradation and so on. However, and a mature and unified understanding of the effect of density and confining pressure on the shear properties of calcareous sand has not been formed; compared with quartz sand, this makes it difficult to provide practical projects with reasonable and effective guidance. Therefore, it is necessary to study the effect of density and confining pressure on the shear properties of calcareous sand.

**Figure 1:**
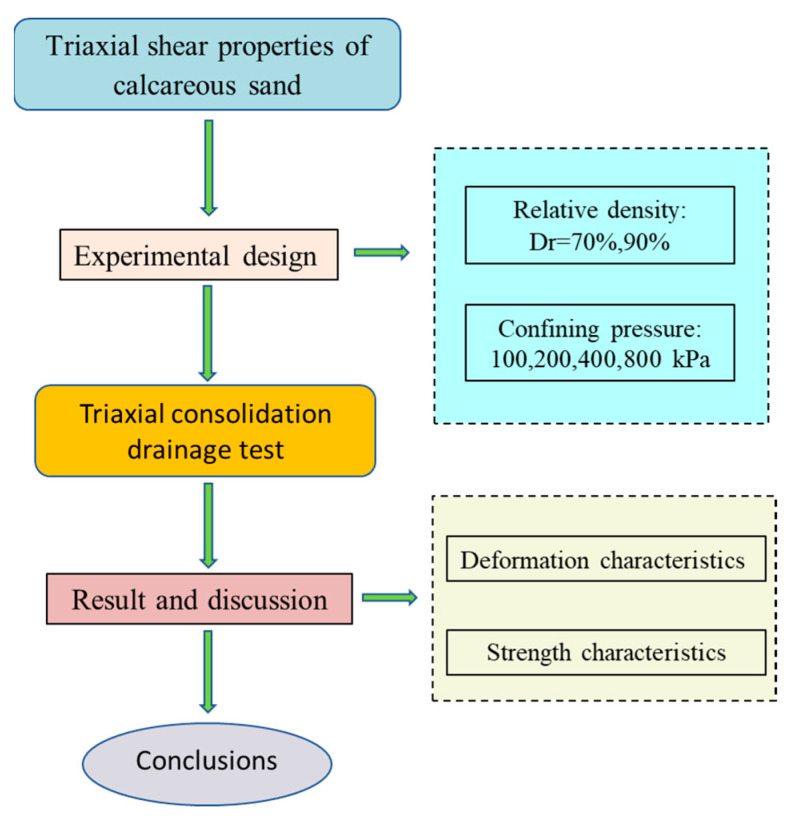
Flow chart for methodology.

In this paper, the triaxial consolidation drainage shear tests were carried out on two sets of calcareous sand samples with relative densities of 70% and 90% under the confining pressures of 100, 200, 400, and 800 kPa. The effects of relative density and confining pressure on deformation and strength characteristics were analyzed. A flow chart for the methodology of this paper in provided in Figure 1. The triaxial shear characteristics of calcareous sand are further understood, which can provide valuable reference for island and reef foundation construction.

## 2. Materials and Methods

### 2.1. Calcareous Sand

**Figure 2:**
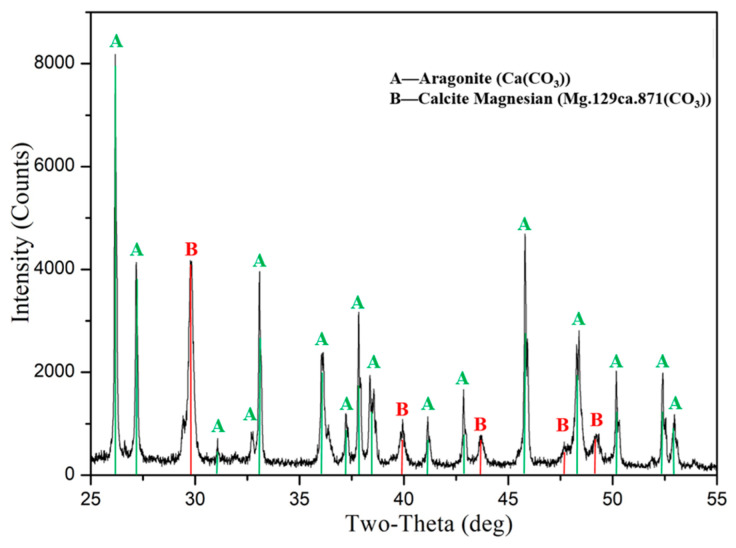
Diffraction pattern of calcareous sand.

**Figure 3:**
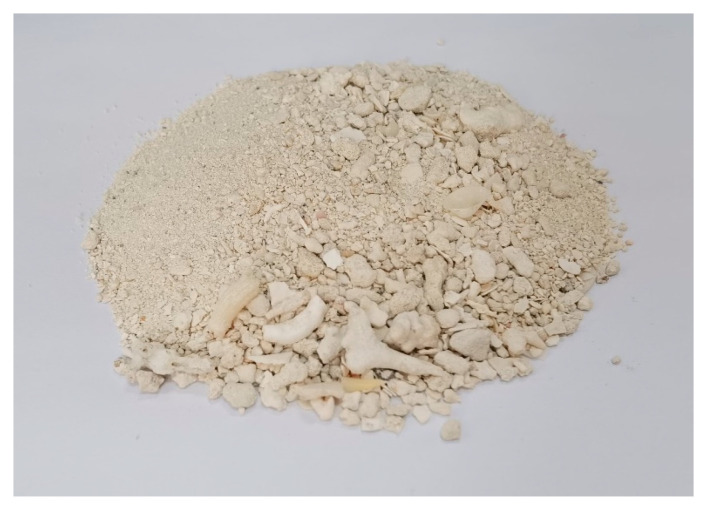
Calcareous sand sample.

The content of CaCO_3_ is the main index that distinguishes calcareous sand from general terrigenous sand. According to international standards, it is classed as calcareous soil when the content of CaCO_3_ in marine soil is more than 50% [28]. Before the experiment, an X-ray diffractometer (XRD) was adopted to obtain characteristics of chemical compositions of calcareous sand, and the diffraction pattern is shown in Figure 2. Its mineral composition is mainly aragonite (CaCO_3_), and it also contains a small amount of magnesian calcite (Mg.129ca.871 (CO_3_)) and other trace impurities. Meanwhile, a large number of coral amputated limbs, shellfish and other biological debris were observed in the sample (Figure 3), which has the genetic characteristics of marine bioclasts, and it is veritable calcareous sand.

**Figure 4:**
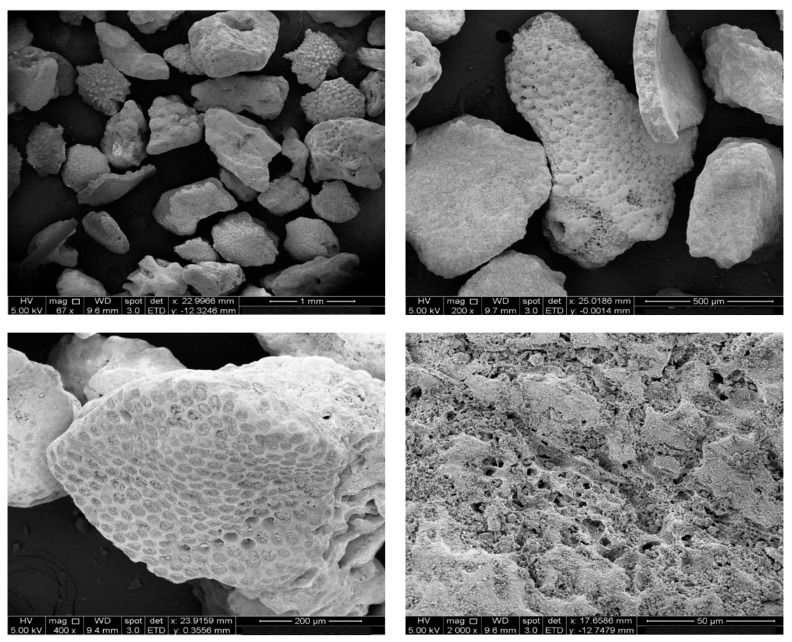
SEM morphology of calcareous sand.

The typical SEM morphology of calcareous sand particles was obtained by electron microscope, as shown in Figure 4. It can be found that calcareous sand particles contain many internal pores, some of which are uniform and dense with deep grooves, and some pores are honeycomb, filled with biological detritus. Additionally, they show irregular shape and high angularity, which is because the calcareous sand has not been transported for a long distance in the process of sedimentation, and retains the structure of the primary biological skeleton.

The maximum and minimum dry densities of calcareous sand are 1.536 and 1.183 g/cm^3^, and the specific gravity is 2.78. The maximum and minimum void ratios of calcareous sand are 1.48 and 1.00, respectively, according to Equation (1).

(1) $$e_{\max}=\frac{G_{s}\rho_{w}}{\rho_{d\min}}-1,\;e_{\min}=\frac{G_{s}\rho_{w}}{\rho_{d\max}}-1,$$

where $G_{s}$ is the specific gravity of calcareous sand, $\rho_{w}$ is the density of water and $\rho_{d\max}$ and $\rho_{d\min}$ are the maximum and minimum dry density, respectively.

**Figure 5:**
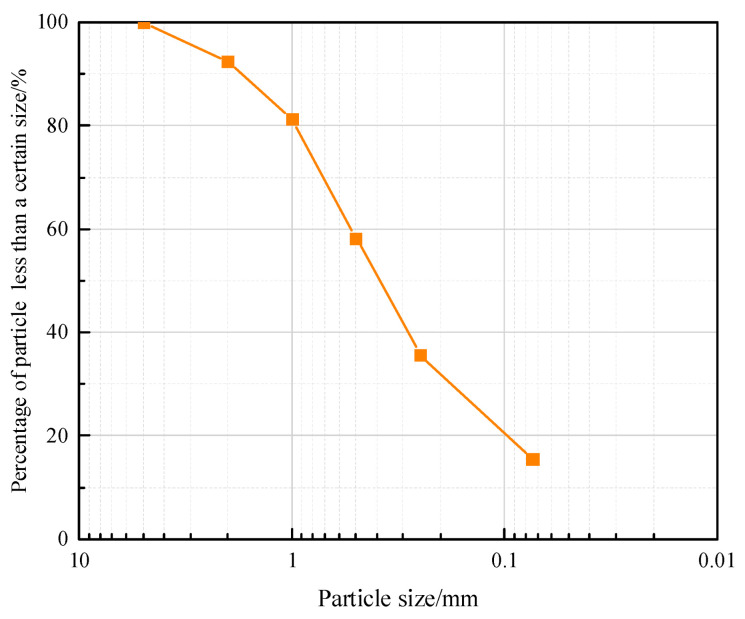
Grading curve of calcareous sand.

The calcareous sand was dried and the particles larger than 5 mm in the specimen were removed. The gradation curve was obtained according to the Geotechnical Test method Standard (GB/T50123-2019), as shown in Figure 5. It can be known that the average particle size of calcareous sand is 0.43 mm, the non-uniformity coefficient is 10.10 and the curvature coefficient is 1.29. It belongs to non-uniform medium sand.

### 2.2. Test Methods

**Figure 6:**
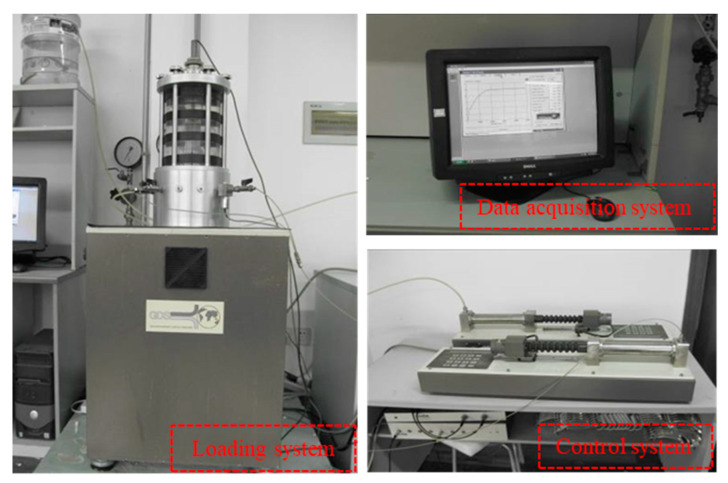
Large-scale triaxial apparatus.

**Figure 7:**
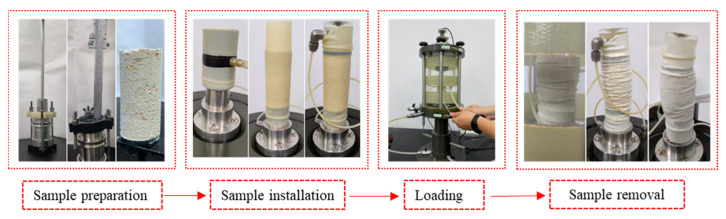
Schematic diagram of triaxial consolidation drainage test process.

Calcareous sand has good water permeability; the water between particles of saturated calcareous sand in the natural environment can dissipate quickly under the action of external force. The stress state of calcareous sand in engineering can be simulated by a consolidation drainage shear (CD) test in the laboratory. The relative density of calcareous sand was designed as Dr = 70% and Dr = 90%; for it to generally reach a compact state after foundation treatment, the confining pressure was set as 100, 200, 400 and 800 kPa, and two parallel tests were designed for each group. According to the design density, calcareous sand samples were prepared by the dry method. Calcareous sand particles are angular and rich in internal pores, so the sample was saturated by a combination of various methods. It was first pumped through CO_2_ to replace the air in it, then saturated with water head, and finally, the back pressure was applied to saturate the sample. The sample is considered completely saturated when the saturation is greater than 95%. The equipment adopted in this experiment is shown in Figure 6. The displacement was controlled by the precision displacement sensor installed on the base with a shear rate of 0.02 mm/min, and the test was stopped when the axial strain reached 20%. The experimental process is shown in Figure 7.

## 3. Results

### 3.1. Stress–Strain Curve

**Figure 8:**
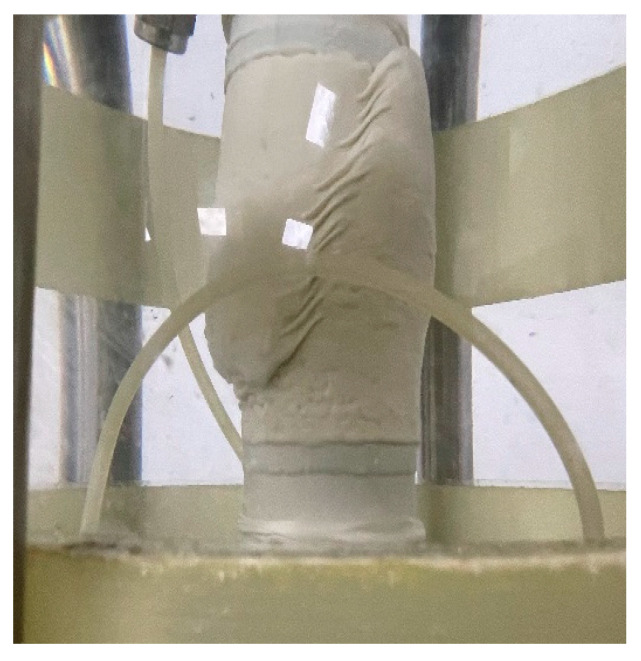
Failure pattern of calcareous sand specimens after consolidation drainage shear.

**Figure 9:**
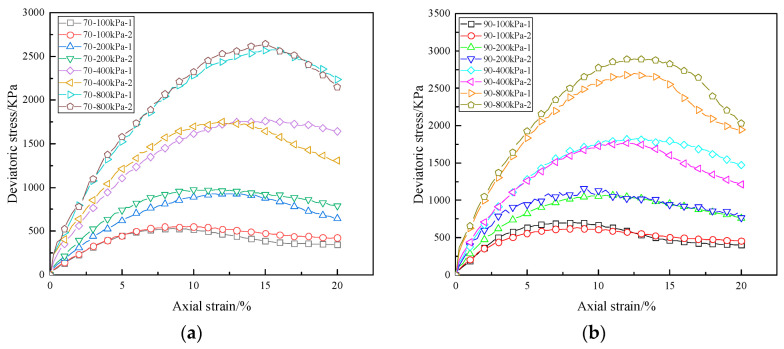
Stress–strain curve of consolidated drained shear for calcareous sand: (**a**) Dr = 70%; (**b**) Dr = 90%.

Shear failure modes generally include bulging and splitting in the triaxial test. In this paper, obvious shear failure zones can be observed after the completion of the consolidated drainage shear of calcareous sand, so the failure mode is splitting failure, as shown in Figure 8. The deviatoric stress and axial strain curves (hereinafter referred to as stress–strain curves) of calcareous sand with relative densities of 70% and 90% under different confining pressures are shown in Figure 9.

As can be seen in Figure 9, the stress–strain curves of the two calcareous sand samples in each test are almost close, and the test results are reliable. The peak deviatoric stress and failure strain of the calcareous sand samples with relative densities of 70% and 90% is smaller under lower confining pressure, and both of them increase with increasing confining pressure. The stress–strain curve under different confining pressure shows a strain softening characteristic. Additionally, the post-peak curve decreases slightly with an unobvious softening characteristic under the lower confining pressure, but the softening characteristic increases gradually with increasing confining pressure. This is because the relative density of the calcareous sand sample in this study is larger, and the sand has formed a relatively stable structure. Compared with the sand samples with low relative density, it has a short compaction process, the stress reaches the peak value quickly and is destroyed, and then the strength begins to decrease gradually. Therefore, the softening phenomenon of the sample with high relative density is more obvious.

**Figure 10:**
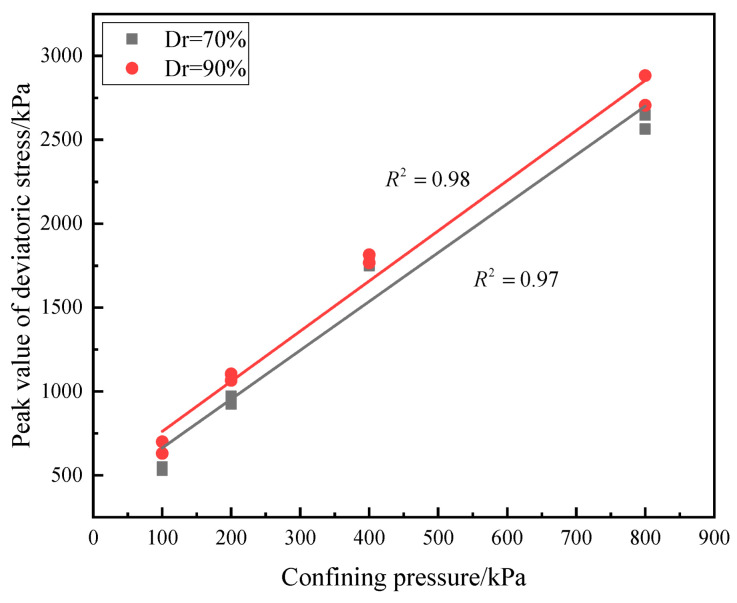
Relationship between peak deviating stress and confining pressure.

**Figure 11:**
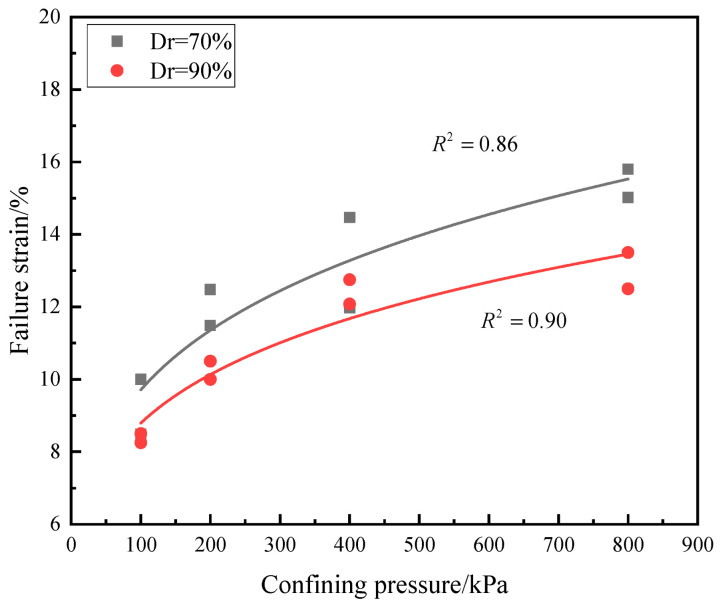
Relationship between failure strain and confining pressure.

The peak values of deviatoric stress and failure strain are shown in Table 1. Additionally, the relationship between the peak value of deviatoric stress and confining pressure is exhibited in Figure 10. It can be seen that the peak values of deviatoric stress of calcareous sand with different relative densities increase linearly with increasing confining pressure. The relationship between failure strain and confining pressure is shown in Figure 11; the failure strain is 10~15.02% for calcareous sand with a relative density of 70%, larger than 8.50~13.50% for that of 90%. The higher the relative density is, the more calcareous sand particles are needed with a constant volume of the sample, and the particles of the sample are arranged more closely. During shearing, the sliding of calcareous sand particles in the sample becomes more and more difficult. Further analysis also found that the failure strain increases by 40~50% when confining pressure increases from 100 kPa to 400 kPa and 4~10% when confining pressure increases from 400 kPa to 800 kPa, and the increase in failure strain is restrained when the confining pressure reaches 400 kPa.

**Figure 12:**
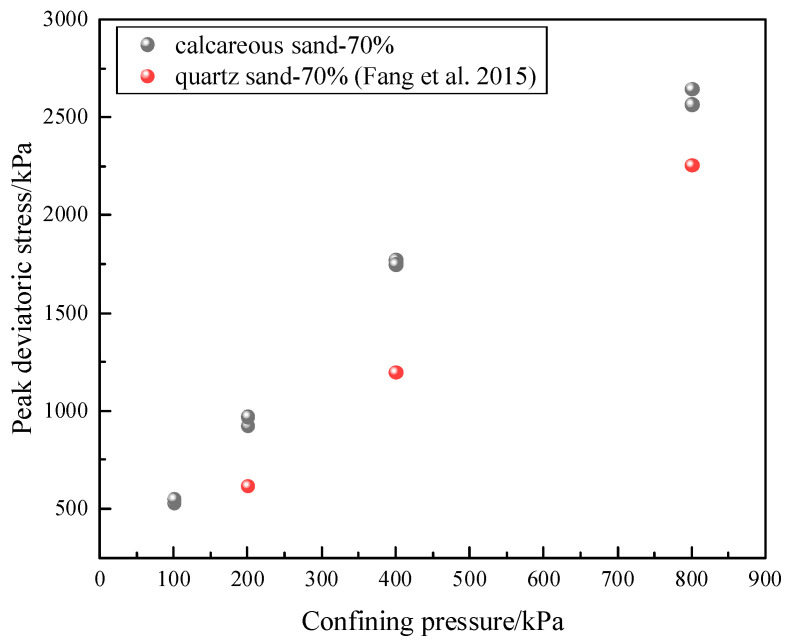
Strength comparison between coral sand and quartz sand [28].

The peak deviatoric stress of calcareous sand is greater than that of quartz sand under nearly the same condition. Take calcareous sand with a relative density of 70% as an example; the peak deviatoric stress is compared with quartz sand [29] with the same relative density, as shown in Figure 12. Additionally, the same findings are also found in previous studies [30,31]. This may be due to the higher interlockings between calcareous sand with more irregular particle shapes than quartz sand. The failure strain of calcareous sand is 8~15%, which is also larger than the 4~8% of ordinary quartz sand [30]. This is because calcareous sand has a high content of CaCO_3,_ usually more than 80%. The Mohs hardness of aragonite is 3.5~4 and that of quartz is 7 [32]. Therefore, calcareous sand with a high content of CaCO_3_ is brittle, with a lower strength and a larger failure strain.

### 3.2. Initial Shear Modulus

Shear modulus is an important parameter to study the material shear properties, which represents the ability of material to resist shear deformation. The relationship between the initial shear modulus $G_{i}$ and the initial tangent modulus $E_{i}$ is shown in Equation (2). In the Duncan–Chang model [33], the relationship of stress–strain is expressed as Equation (3), and the initial tangent modulus is defined as the initial slope of the stress–strain curve, as shown in Equation (4). The initial shear modulus of calcareous sand acquired by (2)~(4) is provided in Table 2.

(2) $$G_{i}=\frac{E_{i}}{2\left(1+v\right)},$$

where $G_{i}$ is the initial shear modulus, $E_{i}$ is the initial tangent modulus and v is Poisson’s ratio, generally taken as 0.3 for calcareous sand.

(3) $$\frac{\varepsilon }{\left(\sigma_{1}-\sigma_{3}\right)}=a+b\varepsilon ,$$

(4) $$E_{i}={\frac{d\left(\sigma_{1}-\sigma_{3}\right)}{d\left(\varepsilon \right)}|}_{\varepsilon \to 0},$$

**Figure 13:**
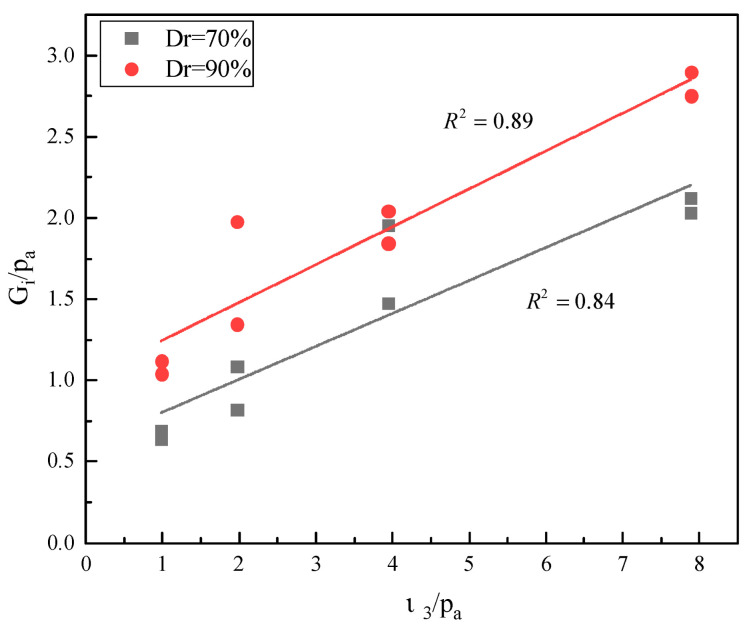
Relationship between initial shear modulus and confining pressure.

The initial shear modulus and confining pressure were converted into dimensionless by standard atmospheric pressure $p_{a}$ (101.4 kPa), and the relationship between initial shear modulus and confining pressure is shown in Figure 13. It was found that the initial tangent modulus increases with the increase in confining pressure; the relationship can be expressed as straight line, as shown in Equation (5). The parameters of calcareous sand with relative densities of 70% and 90% were obtained; that is, $\alpha_{1}$ = 0.20, $\beta_{1}$ = 0.60, $\alpha_{2}$ = 0.23 and $\beta_{2}$ = 1.01. The slope of the two straight lines have little difference, which can be approximately considered as parallel.

(5) $$G_{i}/p_{a}=\alpha_{1}\left(\sigma_{3}/p_{a}\right)+\beta_{1},$$

where α and β are material parameters.

### 3.3. Shear Strength

Shear strength is one of the important mechanical properties for soil; it can reflect the ability of soil to resist shear sliding. Additionally, the two important indices of shear strength are cohesion and internal friction angle. It is widely known that the concept of cohesion is accepted for clayey soil; sand usually has no cohesion. However, numerous studies have reported that calcareous sand has cohesion; it is formed by the bite force between particles, which is called quasi-cohesion.

**Figure 14:**
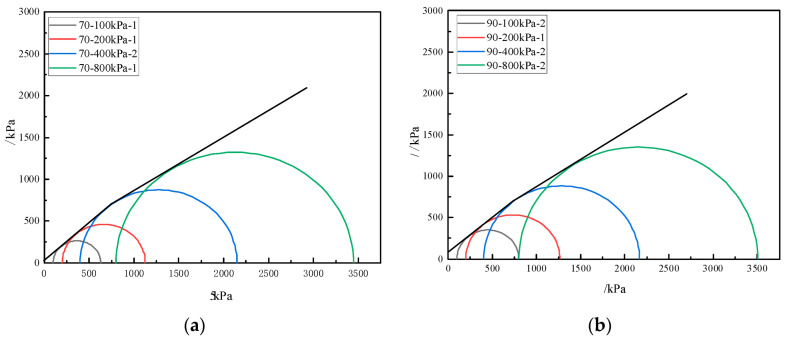
Mohr circle and strength envelope of calcareous sand: (**a**) Dr = 70%; (**b**) Dr = 90%.

The molar circle and strength envelope of calcareous sand under different relative densities is shown in Figure 14 (the strength discreteness of calcareous sand is small and only one group was selected for analysis). It shows that the strength envelope is linear within 100~400 kPa of confining pressure. When it exceeds 400 kPa, the strength envelope begins to deviate from the linear development. Meanwhile, in the range of 100~400 kPa, calcareous sand with a relative density of 70% has a quasi-cohesion of 29.67 kPa and an internal friction angle of 41.88°, while calcareous sand with a relative density of 90% has a cohesion of 81.73 kPa and an internal friction angle of 39.81°. Additionally, the difference in cohesion and internal friction angle for calcareous sand with relative densities of 70% and 90% are 47.8% and 4.9%, respectively. Evidently, the influence of relative density on cohesion is much greater than that on the internal friction angle. The greater the relative density, the smaller the movement space between the calcareous sand particles, and the bite force between the particles is enhanced. When the confining pressure is larger than 400 kPa, the internal friction angle decreases and the cohesion increases slightly. The shear strength characteristics of calcareous sand are quite different from those of ordinary terrestrial sand.

### 3.4. Stress Ratio

Stress ratio can be obtained by dividing the deviatoric stress q by the mean stress p.

**Figure 15:**
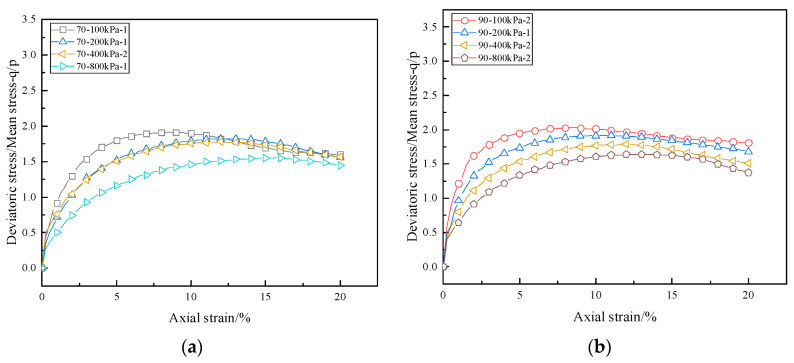
Relationship between stress ratio and strain of calcareous sand: (**a**) Dr = 70%; (**b**) Dr = 90%.

The expressions of deviatoric stress q and mean stress p are specified in Equations (6) and (7). The relationship between the stress ratio q/p and the axial strain of calcareous sand under different confining pressures is illustrated in Figure 15; the stress ratio increases rapidly and reaches the peak stress ratio when the strain is small. As the strain continues to increase, the stress ratio decreases slowly. The ${\left(q/p\right)}_{\max}$ decreases with increasing confining pressure for calcareous sand with a constant relative density, which is opposite to that of the stress–strain in Figure 9. The value of ${\left(q/p\right)}_{\max}$ is 1.63~2.03 for calcareous sand with a relative density of 90%, which is greater than the 1.57~1.91 for calcareous sand of 70%. Additionally, the stress ratio almost converges on a horizontal line with a value of about 1.5 for 70% calcareous sand and about 1.6 for 90% calcareous sand at the final shear stage.

(6) $$q=\sigma_{1}-\sigma_{3},$$

(7) $$p=\frac{1}{3}\left(\sigma_{1}+2\sigma_{2}\right),$$

where $\sigma_{1}$ is the axial stress and $\sigma_{3}$ is the radial stress.

### 3.5. Stress Path

The change in stress state in the specimen in the process of shear can be reflected by the stress path on a particular plane. For the same type of sand, the process of stress change is different when different test methods and loading methods are adopted during the triaxial shear failure, and the deformation and strength characteristics also exhibit great difference. Therefore, the failure process of soil and physical meaning can be analyzed according to the properties of the stress path [34].

**Figure 16:**
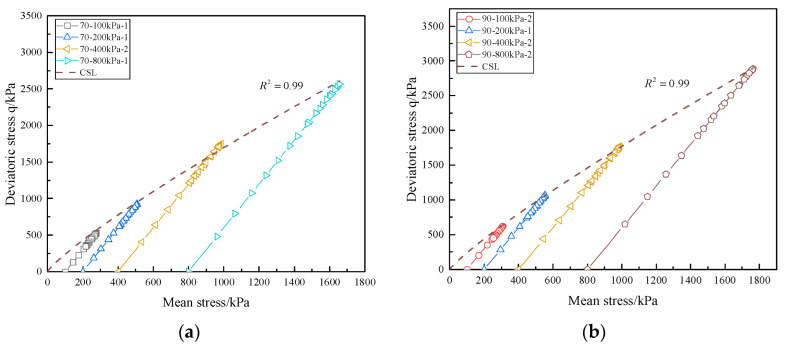
Stress path and critical state line of triaxial consolidated drainage shear: (**a**) Dr = 70%; (**b**) Dr = 90%.

The stress path and CSL of calcareous sand are presented in Figure 16. The stress paths of calcareous sand under different confining pressures are parallel to each other, with a constant stress ratio of dq/dp=3. However, the length of the stress path increases with increasing confining pressure, and the higher the relative density is, the longer the stress path is. Due to the softening phenomenon of calcareous sand, the stress path first extends along the line to the peak state, and then turns back. The critical state theory points out that the soil will eventually reach a limit state with the development of shear strain. When the sand is in the limit state, the shear stress and void ratio are constant, but the shear strain is still changing continuously, which is called the critical state [35]. The CSL can be determined by the triaxial shear failure point [36]. The CSL of ordinary terrestrial sand is a straight line passing through the origin, which is usually expressed by Equation (8), but the CSL of calcareous sand under different confining pressures shows nonlinear properties, which can be fitted by Equation (9). The parameters are $M_{1}=4.70$, N=0.85 for calcareous sand of 70% and $M_{1}=4.47$, N=0.87 for calcareous sand of 90%; the parameters of different relative densities are almost close. In classical soil mechanics, it is considered that the sand particles are not compressed and broken, and the CSL in the stress space is only one straight line, but the particles of calcareous sand are easy to be broken during shearing, so the CSL is nonlinear.

(8) q=Mp,

where M is stress ratio, *q* is deviatoric stress and *p* is mean stress.

(9) $$q=M_{1}p^{N},$$

where $M_{1}$ and N are constant.

## 4. Discussion

An accurate understanding of the triaxial shear characteristics of calcareous sand is very important for the safety of foundations in island and reef engineering. Some studies [24,37,38] found that the stress–strain curves of calcareous sand with different densities showed obvious strain softening at low confining pressure, but it gradually changed to strain hardening with the increase in confining pressure, which is different from the result in this study. Weng [27] found that there was strain softening in the range of small particle size (0.075~0.25 mm), but the degree of strain softening decreased with the increase in confining pressure. With the increase in particle size, strain softening gradually changed to strain hardening. The type of the stress–strain curves is affected by confining pressure and the particle size of calcareous sand. The gradation of calcareous sand in this study is quite different from that in references [24,37,38], and both the non-uniformity coefficient and the curvature coefficient are larger. The shear failure pattern of calcareous sand in this paper is consistent with that in the literature [30], both of which formed obvious shear failure zones. The deviatoric stress of calcareous sand reaches the peak value, then brittle failure occurs and the deviatoric stress decreases obviously with the increase in strain, showing the characteristic of strain softening, which is in accordance with the softening characteristic of the stress–strain curve.

The Molar circle envelope of calcareous sand is linear within the range of 100~400 kPa, but it deviates from the linear in the range of 400~800 kPa, which is different from the single linearity in the literature [24,30]. Because the calcareous sand in this paper has a large non-uniform coefficient, when the confining pressure reaches 400 kPa, a large number of calcareous sand particles begin to break, resulting in a change in the slope of the Mohr circle envelope.

However, there are limitations in this study; no more control group experiments on confining pressure, density and different sand materials have been carried out. Additionally, calcareous sand particles have irregular shapes; in addition to confining pressure and relative density, the particle gradation and particle morphology also have great influence on the mechanical properties of calcareous sand, but no further study has been performed.

## 5. Conclusions

Calcareous sand is widely used in the foundation construction and engineering of islands and reefs, and it exhibits special mechanical properties due to its irregular particle shape and high void ratio, and the fact that it is easily broken. In order to investigate the shear properties of calcareous sand, triaxial consolidation drainage shear tests on calcareous sand samples with relative densities of 70% and 90% under confining pressures of 100, 200, 400 and 800 kPa were conducted, and the effects of relative density and confining pressure on the deformation and strength characteristics of calcareous sand were analyzed. The main conclusions are as follows:

1. The stress–strain curves of calcareous sand show a strain softening characteristic, which was enhanced with the increasing confining pressure. The increment in failure strain from 100 kPa to 400 kPa is much larger than that from 400 kPa to 800 kPa, and it is restrained when confining pressure reaches 400 kPa.
2. The initial shear modulus of calcareous sand with relative densities of 70% and 90% increases linearly with almost the same slope as the increasing confining pressure.
3. The envelope of calcareous sand’s molar circle is linear within the range of 100~400 kPa, but it deviates from the linear in the range of 400~800 kPa. In the linear stage, the cohesion and internal friction angle of calcareous sand with relative densities of 70% and 90% are 93.60 kPa, 36.55° and 118.04 kPa, 36.75°, respectively.
4. The peak stress ratio of calcareous sand decreases with increasing confining pressure. The stress ratio in the final shear stage of the test almost converges on a horizontal line with the value of about 1.5 for 70% calcareous sand and about 1.6 for 90% calcareous sand.
5. The CSL of calcareous sand exhibits non-linearity, which can be described by a power exponential function with almost the same exponent for calcareous sand with different relative densities.

## Figures and Tables

**Table 1:** Peak deviatoric stress and failure strain of calcareous sand.

Test No.	Relative Density/%	Confining Pressure/kPa	Peak Deviatoric Stress/kPa	Failure Strain/%
CD70-100-1	70	100	529.85	8.49
CD70-100-2		100	551.00	9.28
CD70-200-1		200	925.30	12.48
CD70-200-2		200	971.59	11.49
CD70-400-1		400	1771.36	11.97
CD70-400-1		400	1750.66	14.47
CD70-800-1		800	2565.01	15.02
CD70-800-1		800	2646.86	15.80
CD90-100-1	90	100	700.05	8.25
CD90-100-2		100	630.61	8.50
CD90-200-1		200	1131.95	10.50
CD90-200-2		200	1066.29	10.00
CD90-400-1		400	1824.56	12.08
CD90-400-1		400	1766.40	12.75
CD90-800-1		800	2697.40	12.50
CD90-800-1		800	2888.47	13.50

**Table 2:** Initial shear modulus.

Test No.	$G_{i}/\mathrm{kPa}$	Test No.	$G_{i}/\mathrm{kPa}$
70-100kPa-1	64.75	90-100kPa-1	105.37
70-100kPa-2	70.18	90-100kPa-2	113.46
70-200kPa-1	82.89	90-200kPa-1	136.39
70-200kPa-2	109.89	90-200kPa-2	200.32
70-400kPa-1	149.65	90-400kPa-1	186.71
70-400kPa-2	198.25	90-400kPa-2	206.78
70-800kPa-1	205.68	90-800kPa-1	278.71
70-800kPa-2	214.87	90-800kPa-2	293.60

## Data Availability

The data presented in this study are available on request from the corresponding author.
